# Molecular Mechanisms and Crosstalk Signaling in Soybean’s Response to Water Deficit and Excess: Implications for Stress Resilience and Productivity

**DOI:** 10.3390/plants14213245

**Published:** 2025-10-23

**Authors:** Elizandra Carneiro Andreata, Mayla Daiane Correa Molinari, João Matheus Kafer, Silvana Regina Rockenbach Marin, Daniel Rockenbach Marin, Renata Fuganti-Pagliarini, André Luis Laforga Vanzela, Elibio Leopoldo Rech, Alexandre Lima Nepomuceno, Liliane Marcia Mertz-Henning

**Affiliations:** 1 Department of General Biology, State University of Londrina, Londrina 86057-970, Paraná, Brazil; elizandra.andeatta12@gmail.com (E.C.A.); andrevanzela@uel.br (A.L.L.V.); alexandre.nepomuceno@embrapa.br (A.L.N.); liliane.henning@embrapa.br (L.M.M.-H.); 2 Embrapa Soybean, Londrina 86085-981, Paraná, Brazil; silvana.marin@embrapa.br (S.R.R.M.); dani.rockenbachmarin@gmail.com (D.R.M.); 3 Sempre Agtech, Chapecó 89802-210, Santa Catarina, Brazil; mdcmolinari@gmail.com; 4 Department of Exact Sciences, State University of Londrina, Londrina 86057-970, Paraná, Brazil; renatafuganti@gmail.com; 5 Picolla Scientific Consulting, Saskatoon, SK S7W 1B7, Canada; 6 Embrapa Genetic Resources and Biotechnology, Brasília 70770-917, Goiás, Brazil; elibio.rech@embrapa.br

**Keywords:** *Glycine max*, drought stress, flood stress, gene expression, abscisic acid (ABA), crosstalk signaling

## Abstract

Soybean plays a crucial role in global food security and the economy, but its yield is often limited by water deficit (WD) and water excess (WE). Understanding the molecular mechanisms that regulate responses to these stresses is essential to improve crop resilience. In this study, we analyzed nine ABA-induced genes involved in WD and WE signaling using transcriptome libraries, RT-qPCR, gas exchange analysis, and root morphology. A total of 4412 and 2597 genes were differentially expressed under WE and WD, respectively. The response to exogenous ABA varied between conditions, reflecting stress-specific adaptations. Among 10 genes exclusively expressed under WE, only ERF1 and Peroxydase showed increased transcript levels after ABA treatment, being similarly regulated under both WD and WE. These results reveal distinct molecular and physiological responses to ABA depending on water status, highlighting potential targets for genetic improvement. The identified genes provide insights into ABA-mediated regulation of soybean stress tolerance and represent promising candidates for breeding strategies aimed at enhancing resilience to water-related stresses. Ultimately, this study contributes to a deeper understanding of soybean adaptation mechanisms, supporting sustainable crop management and productivity under challenging environmental conditions.

## 1. Introduction

Soybean (*Glycine max* L.) is one of the most important crops worldwide, providing protein and oil for food, feed, and industrial applications. However, its productivity is increasingly challenged by climate change, which intensifies episodes of water deficit (WD) and water excess (WE) [1,2]. These contrasting water conditions profoundly impair soybean physiology and yield stability. Under WD, plants exhibit reduced stomatal conductance, lower leaf water potential, decreased photosynthetic performance, and reduced biomass accumulation [3,4,5]. Conversely, flooding stress disrupts physiological functions, reducing plant height, leaf area, chlorophyll content, nutrient uptake, and biomass, with yield losses of up to 51% in sensitive genotypes [6,7,8].

Abscisic acid (ABA) is a central phytohormone in stress adaptation. Its role in drought responses is well established, regulating stomatal closure, photosynthesis, and stress-related gene expression [9,10,11]. However, under waterlogging, the role of ABA remains less clear. Recent studies suggest its interaction with ethylene, ROS, and other signaling pathways to mitigate hypoxia [12,13,14], but a precise molecular understanding of ABA-regulated genes under WE is still missing.

This knowledge gap is particularly relevant because soybean is frequently exposed to alternating or simultaneous episodes of drought and flooding in the field. To address this limitation, we investigated ABA-responsive genes regulated under WD and WE, integrating transcriptomic analyses with RT-qPCR validation, together with physiological, morphological, and bioinformatic approaches. Our goal was to provide a comprehensive view of ABA-mediated regulation under contrasting water stresses and to highlight consistent candidate genes as potential resources for future functional studies and breeding strategies.

## 2. Results

### 2.1. Gas Exchange Analysis in Leaves of Williams 82 Soybean Cultivar

**Figure 1:**
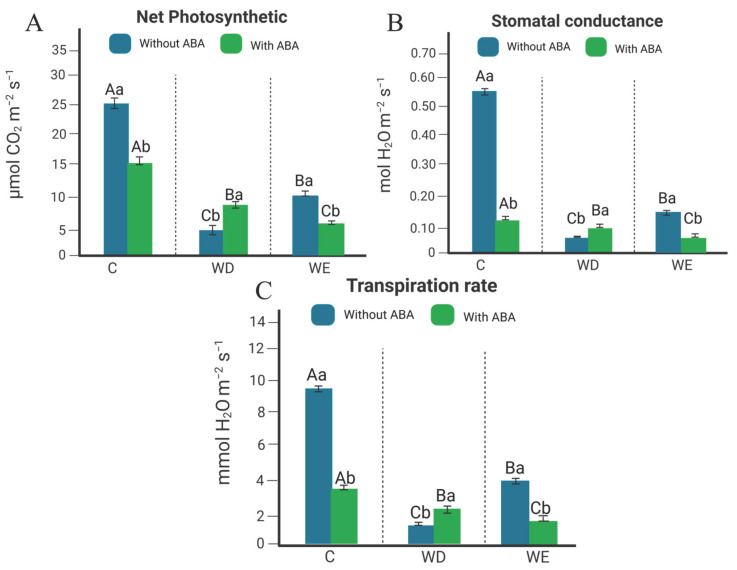
Soybean plants of Williams 82 cultivar, with and without ABA application, subjected to water deficit (WD), water excess (WE), and control (C) conditions. In (**A**) Stomatal conductance—gs; (**B**) Net Photosynthesis; and (**C**) Transpiration rate—E. Uppercase letters compare treatments with and without ABA application separately. Lowercase letters compare ABA application within each water treatment. Mean comparison was performed using Tukey’s test at a significant level of 5%. Error bars represent the standard error of eight biological replicates. Note: mmol = millimoles.

Gas exchange parameters revealed contrasting effects of WD and WE on soybean physiology. In the absence of ABA, both WD and WE significantly reduced stomatal conductance, net photosynthesis, and transpiration, with decreases of ~35–45% compared to control plants (Figure 1A–C). Exogenous ABA application under control conditions also decreased these parameters by ~20%, which is expected since ABA treatment mimics stress signaling, inducing stomatal closure and reduced CO_2_ assimilation even in non-stressed plants. When combined with water stress, however, distinct interaction patterns were observed. Under WD + ABA, stomatal conductance, net photosynthesis, and transpiration increased by approximately 25% relative to WD alone, suggesting a protective effect of ABA in mitigating drought-induced limitations. In contrast, under WE + ABA, all three parameters declined an additional 15–20% compared with WE alone, indicating that ABA exacerbates the negative effects of waterlogging on gas exchange. These quantitative patterns reinforce the dual and context-dependent role of ABA in soybean physiology.

### 2.2. Root Morphology

**Figure 2:**
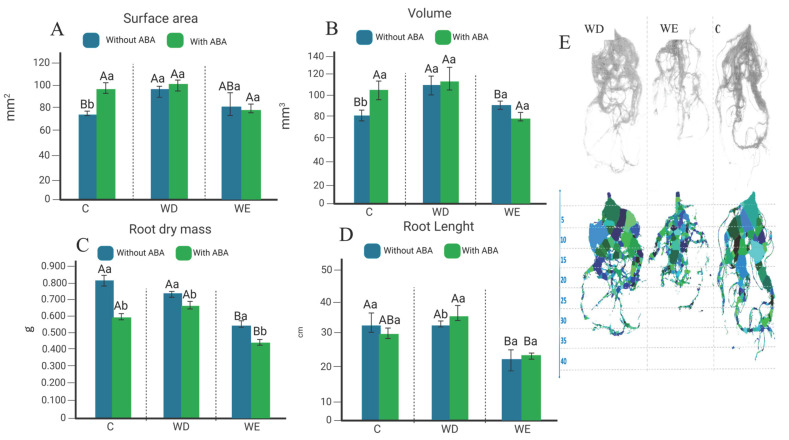
Morphological parameters of Glycine max (Williams 82) roots with and without ABA under water deficit (WD), water excess (WE), and control (C) conditions: (**A**) surface area (mm^2^), (**B**) volume (mm^3^), (**C**) root dry mass (g), and (**D**) root length (cm). Uppercase letters compare treatments with and without ABA separately; lowercase letters compare ABA application within each water condition. Means were compared by Tukey’s test (5% significance), with error bars showing the standard error of eight biological replicates. (**E**) scanned root images for WD, WE, and C without ABA, with root length representing the average per treatment. Root segmentation and diameter classification were automatically performed by the Safira software, which assigns different colors to root segments according to their diameter classes.

Root system responses also differed across treatments (Figure 2). Under WE without ABA, a slight increase in root dry mass was observed, while surface area, volume, and length remained unchanged. In contrast, exogenous ABA application in control conditions reduced root dry mass but promoted greater surface area and volume. This response is expected, as ABA application in non-stressed plants mimics stress signaling, triggering architectural adjustments that favor root system expansion at the expense of biomass accumulation. Under WD, root traits also responded dynamically: in the absence of ABA, surface area and volume increased, whereas ABA application reduced dry mass but significantly increased root length, indicating a compensatory strategy to improve soil exploration under drought. Under WE + ABA, root dry mass decreased further, but ABA partially restored root volume, suggesting that hormonal signaling can counteract some aspects of hypoxia-induced inhibition, although at the cost of biomass accumulation. Altogether, these results demonstrate that ABA modulates root traits in a context-dependent manner, enhancing plasticity and compensatory growth under drought, but limiting root biomass and amplifying constraints under flooding conditions.

### 2.3. Soybean Differentially Expressed Genes Under WD and WE Conditions

**Figure 3:**
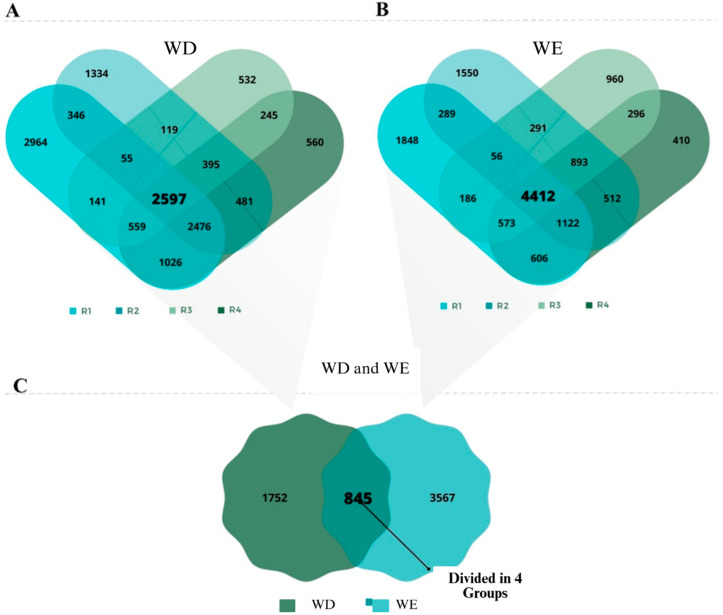
In (**A**) Differential gene expression in water deficit (WD) and in (**B**) water excess (WE) conditions analyzed using Gfold v1.1.4 and EdgeR v3.11 softwares on RNA-Seq libraries. Differentially expressed genes were identified based on a Log2FoldChange (Log2Fc) ex-pression threshold of ≥1 and ≤−1. (**C**) Genes exhibiting differential expression in both treatments. Biological replicates R1, R2, and R3 were analyzed individually using Gfold, and then these three replicates were analyzed together using EdgeR, forming replicate R4. This dual-software approach was used to increase the robustness and.

Aiming to increase the robustness and accuracy of gene selection, we combined two complementary algorithms, GFold and EdgeR, to analyze differential gene expression under water deficit (WD) and water excess (WE). GFold independently analyzed each biological replicate, capturing unique variations across samples and identifying 10,222, 7847, and 4670 differentially expressed genes in WD replicates R1, R2, and R3, respectively. In contrast, EdgeR considered the biological variation among replicates, identifying 8379 differential genes (R4). By intersecting results from both approaches, 2597 genes were consistently detected as differentially expressed under WD (Figure 3A). A similar pattern was observed for WE, with GFold identifying 9145, 9179, and 7709 genes across the three replicates, while EdgeR identified 8869; comparison between methods resulted in 4412 genes consistently regulated under WE (Figure 3B). Importantly, 845 genes were common to both WD and WE (Figure 3C). These were classified into four groups: Group 1, genes upregulated under both stresses (368 genes); Group 2, downregulated in both stresses (423 genes); Group 3, upregulated in WD but downregulated in WE (36 genes); and Group 4, downregulated in WD but upregulated in WE (17 genes) (Supplementary File S2).

This filtering strategy ensured that only genes with consistent regulation across algorithms and replicates were retained, reducing false positives and strengthening biological interpretation. Moreover, the identification of shared and stress-specific groups already points to distinct regulatory modules: while Groups 1 and 2 likely represent a core stress-responsive network, Groups 3 and 4 highlight genes fine-tuned to contrasting water imbalances. From this dataset, we further refined gene selection for RT-qPCR by prioritizing candidates with more than two ABA-responsive ABRE motifs in their promoters (for WD) and at least one WE-associated motif (ACCCC, GGTTT, or AAACCA). Together, this dual-algorithm and motif-guided approach enhanced the reliability of gene selection and provided a comprehensive framework to interpret transcriptomic responses to water deficit and excess in soybean.

### 2.4. Identification of WD, and WE Responsive Motifs

**Figure 4:**
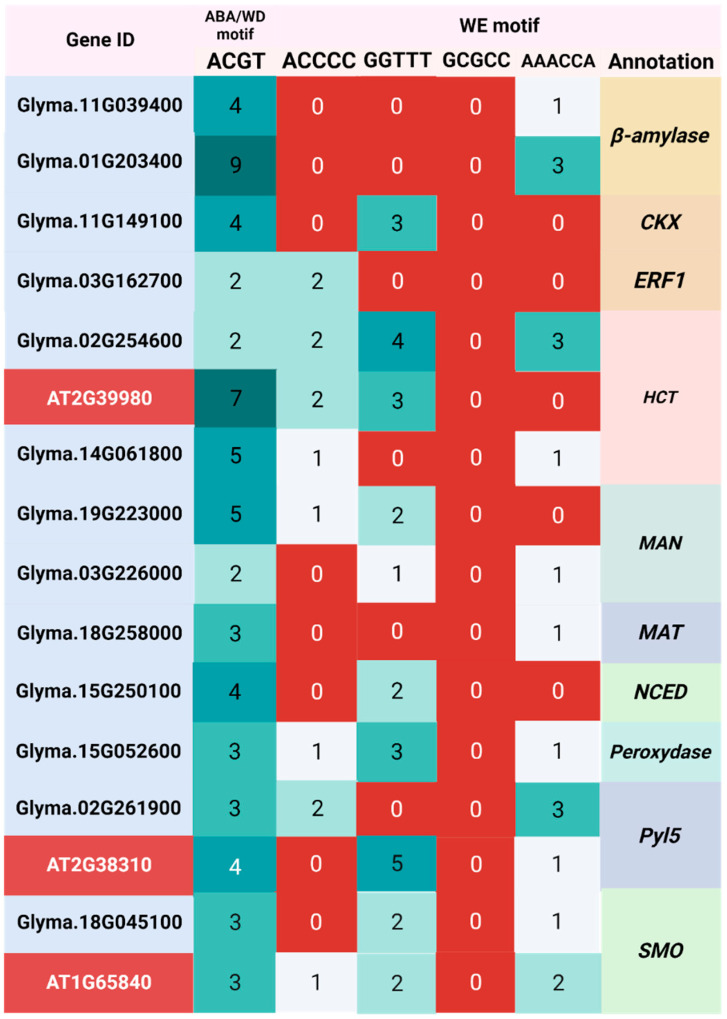
Counts of ABA-responsive, water deficit-responsive (WD), and water excess-responsive (WE) motifs. The counts were obtained from the 2000bp upstream region of the transcription start site (TSS) of the genes selected for RT-qPCR validation, as well as their corresponding copies in soybean and orthologs in Arabidopsis thaliana. The Gene IDs highlighted in red indicate the identification of orthologs in *A. thaliana*.

To further strengthen the biological relevance of gene selection, we analyzed the promoter regions (2000 bp upstream of the transcription start site) of the candidate genes for the presence of stress- and hormone-responsive cis-regulatory motifs. All ten genes selected in silico contained at least two ABA-responsive ABRE motifs (ACGT), confirming their potential regulation by ABA under water deficit (Figure 4). Although none of the genes carried the GCGCC motif, which is specifically associated with flooding responses, all possessed at least one of the other WE-related motifs (ACCCC, GGTTT, or AAACCA). The presence of these motifs suggests that the selected genes are transcriptionally regulated not only by ABA but also by signaling pathways associated with hypoxia and flooding.

This motif-based validation provided an additional layer of evidence supporting the functional involvement of the chosen genes in water stress responses. By combining transcriptomic consistency (detected by both GFold and EdgeR) with promoter motif enrichment, we ensured that the genes selected for RT-qPCR represent robust candidates, mechanistically linked to ABA signaling and the contrasting stresses of WD and WE.

### 2.5. Comparative Analysis of Gene Expression in Response to Water Treatments: Consistent Regulation Patterns Between RNA-Seq and RT-qPCR

**Figure 5:**
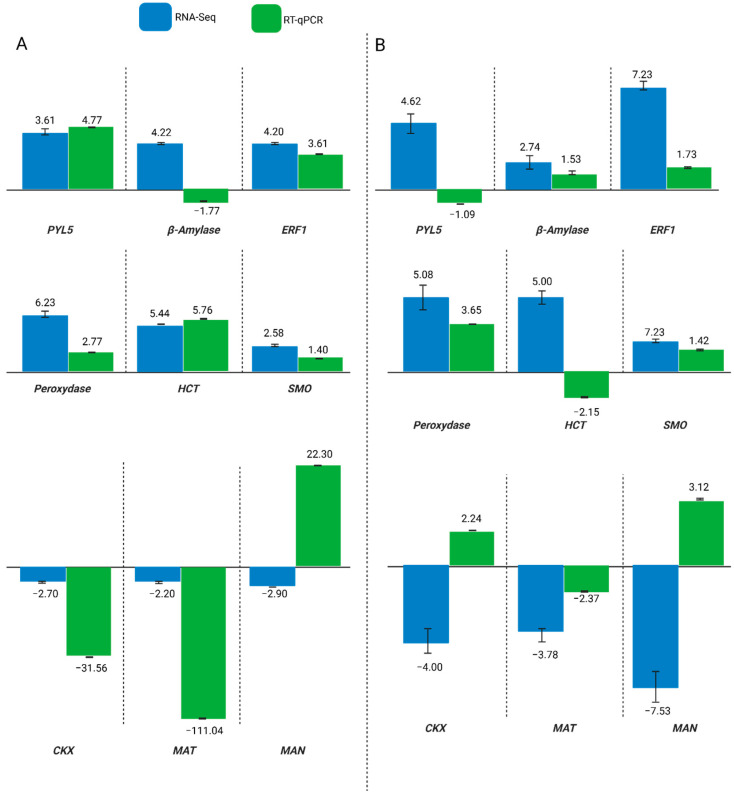
Gene expression in soybean (Williams 82) leaves under water deficit (**A**) and water excess (**B**) was analyzed by RNA-Seq and RT-qPCR. Expression levels were calculated using the 2^−∆∆Ct^ method with primer efficiencies [15], normalized to the water control. Error bars indicate the standard error of three biological replicates. Green bars show RT-qPCR results, and blue bars show RNA-Seq data. RT values correspond to the average log2FoldChange (log2FC) from Gfold and EdgeR analyses.

To validate transcriptomic results and refine candidate gene selection, nine genes were analyzed by RT-qPCR under WD and WE conditions (Figure 5). Under water deficit, 7 of the 9 genes (*PYL5*, *ERF1*, *Peroxydase*, *HCT*, *SMO*, *CKX*, and *MAT*) showed consistent expression patterns between RNA-seq and RT-qPCR, confirming the robustness of their regulation in response to drought. In contrast, under water excess, 5 of the 9 genes (*β-amylase, ERF1, Peroxydase*, *SMO*, and *MAT*) were validated across both techniques. ERF1 and Peroxydase emerged as the most reliable markers, being consistently upregulated under both WD and WE in both datasets, while *SMO* and *MAT* also showed reproducible regulation but in a stress-dependent manner. Three genes (*PYL5*, *HCT*, and *CKX*) were consistent only under WD, highlighting their drought-specific roles, whereas β-amylase was validated only under WE, suggesting a more prominent role in flooding tolerance. One gene (*MAN*) displayed contrasting patterns between RNA-seq (upregulated) and RT-qPCR (downregulated) and was excluded from further analysis.

These results demonstrate that the majority of the selected genes were validated under WD, while fewer showed consistent regulation under WE, reinforcing the idea that drought triggers a more stable ABA-dependent transcriptional response compared to flooding.

### 2.6. Effect of ABA on Gene Expression Under WD and WE Conditions

**Figure 6:**
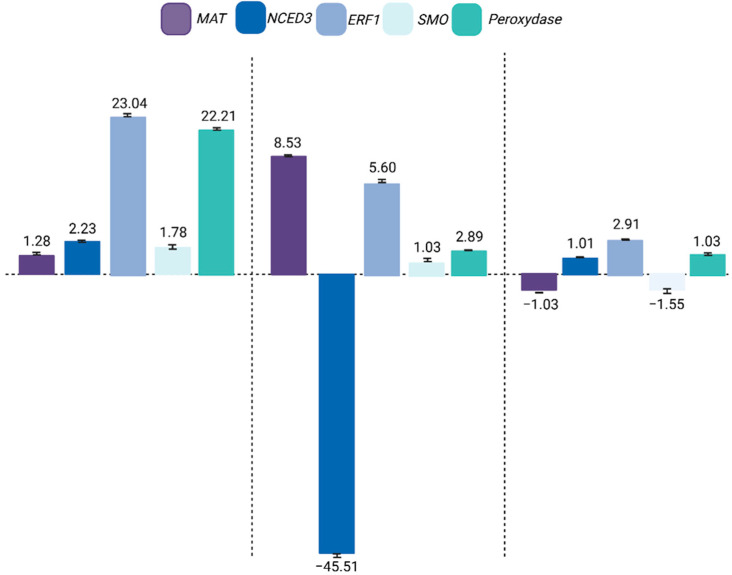
Gene expression of in silico selected and validated genes in soybean (Williams 82) leaves showed consistent regulation in RNA-Seq and RT-qPCR under water deficit (WD) and water excess (WE). Expression was quantified using the Livak method [15] and normalized to the condition without exogenous ABA. Up- and downregulation indicate the effects of ABA application. *NCED3* was used as a reference to confirm endogenous ABA biosynthesis.

**Figure 7:**
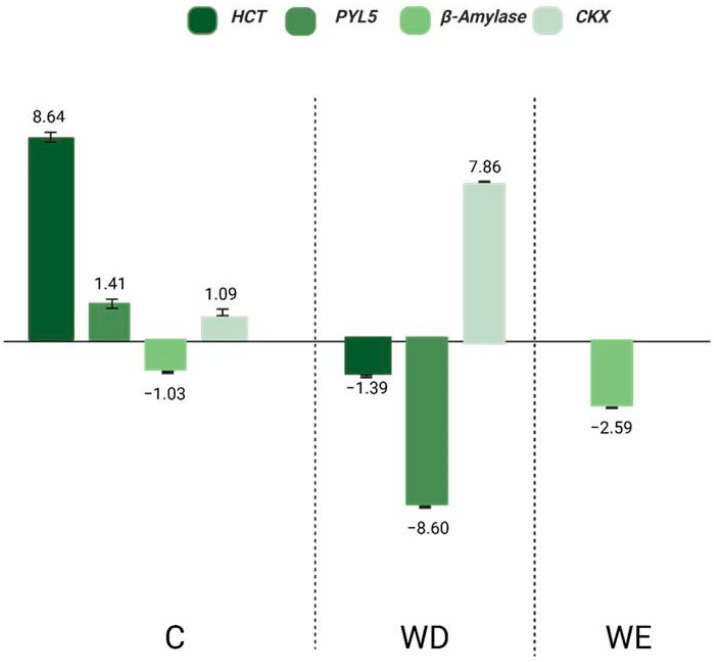
Comparison of gene expression profiles between RNA-Seq and RT-qPCR for selected genes (*HCT*, *PYL5*, β-amylase, and *CKX*) in *Glycine max* (Williams 82) leaves under specific water conditions, showing consistent regulation patterns between techniques. Expression was quantified using the Livak method and normalized to the control without exogenous ABA. Up- and downregulation indicate the effects of ABA application on gene expression.

Given the central role of ABA in stress responses, we next evaluated its effect on gene expression under WD and WE conditions (Figure 6 and Figure 7). Under control conditions, exogenous ABA increased the expression of all five consistently validated genes (*MAT*, *NCED3*, *ERF1*, *SMO*, and *Peroxydase*), confirming that ABA application mimics stress signaling even in the absence of environmental constraints.

In WD + ABA, four of these genes (*MAT*, *ERF1*, *SMO*, and *Peroxydase*) were upregulated, while *NCED3* was downregulated, likely reflecting a feedback mechanism to prevent excessive ABA accumulation when exogenous hormone is supplied. In contrast, under WE with ABA, *MAT* and *SMO* expression decreased, whereas *NCED3, ERF1*, and Peroxydase were induced. This context-dependent regulation highlights the dual role of ABA in soybean: under drought, it enhances protective pathways linked to stomatal regulation, redox balance, and structural adjustment, while under flooding it differentially modulates biosynthesis and signaling genes, amplifying some responses (e.g., *NCED3*, *ERF1*, *Peroxydase*) but suppressing others (e.g., *SMO*, *MAT*).

Notably, *ERF1* and *Peroxydase* were consistently upregulated by ABA under both stresses, underscoring their function as stable downstream targets of ABA signaling. Conversely, *SMO* and *MAT* showed opposite regulation between WD and WE, suggesting that their activity is finely tuned by the interaction of ABA with stress-specific pathways such as ethylene and ROS homeostasis. *NCED3* displayed a clear feedback signature, being repressed in WD + ABA but induced in WE + ABA, further illustrating how ABA biosynthesis itself is context-dependent. Together, these patterns confirm that ABA application does not act uniformly but rather modulates a network of stress-related genes in a stress-specific manner.

### 2.7. Gene Copies, Transcripts, and Orthologs in Soybean (Glycine max) and Arabidopsis thaliana: Insights into Conservation and Functional Implications

To gain further insights into the evolutionary conservation and functional potential of the candidate genes, we analyzed their copy number, transcript variants, and orthologs in soybean and Arabidopsis thaliana (Table 1). Three soybean genes *(MAN*, *HCT*, and *β-amylase*) were present in duplicated copies located on different chromosomes, with protein sequence similarity above 92%, suggesting functional redundancy and potential dosage effects in stress responses. In contrast, the other genes (*NCED3*, *PYL5*, *ERF1*, *SMO*, *MAT*, *CKX*, and *Peroxydase*) were found as single-copy genes, with only two of them displaying alternative transcripts. The limited redundancy of these loci may explain their more stable and consistent regulation across treatments, as observed for ERF1 and Peroxidase.

Comparative analysis also revealed orthologs for three of the soybean genes in Arabidopsis, all existing as single-copy genes with ~70% protein sequence similarity. This degree of conservation indicates that stress-related functions mediated by ABA, ethylene, or ROS signaling may be at least partially shared across species, while still allowing for soybean-specific adaptations. In particular, orthologous relationships for *SMO*, *PYL5*, and *HCT* provide additional confidence that these genes are part of conserved regulatory modules relevant for abiotic stress adaptation.

Altogether, this analysis highlights that while certain genes (e.g., *MAN*, *HCT*, *β-amylase*) may contribute to stress responses through redundant gene copies, others (e.g., *ERF1*, *Peroxydase*, *NCED3*) are maintained as single copies, which reinforces their role as critical, non-redundant regulators. Such information is particularly valuable for translational studies, since conserved genes with single-copy status are often more promising for functional characterization and biotechnological applications aimed at improving stress resilience in soybean and related crops.

## 3. Discussion

### 3.1. Physiological and Transcriptomics Insights into WD and WE Stress

Our findings demonstrate that ABA exerts contrasting effects on soybean physiology depending on the type of water stress. Under WD, exogenous ABA partially restored stomatal conductance, net photosynthesis, and transpiration, whereas under WE, it exacerbated reductions in these parameters (Figure 1). These results are consistent with previous reports showing that ABA enhances drought tolerance by maintaining carbon assimilation and water status [16,17]. Conversely, the negative effect of ABA under WE contrasts with observations in other species. For instance, in maize, exogenous ABA significantly improved survival under anoxia by increasing alcohol dehydrogenase activity [18], while in rice, ABA-related proteins were upregulated during combined low-temperature and flooding stress [19]. Moreover, in soybean, crosstalk between ABA and ethylene signaling under flooding shows that tolerant genotypes have lower ABA levels but higher ethylene, illustrating a complex hormonal interaction [14]. Together, these findings suggest that soybean exhibits a species-specific response to ABA under water excess, and underscore the importance of investigating ABA’s dualistic functions across different crop species.

In addition to its physiological effects, ABA also altered soybean root architecture under control and stress conditions. Specifically, in control plants treated with ABA, we observed a reduction in root dry mass but a simultaneous increase in root surface area (Figure 2). Although ABA treatment in control plants reduced root dry mass, the increase in root surface area likely reflects an architectural adjustment rather than a contradiction. ABA is known to modulate root system structure by influencing lateral root production, elongation, and cell division, acting as a “hidden architect” of root architecture [20]. Furthermore, localized ABA signaling has been shown to mediate root growth plasticity in response to environmental cues [21], and ABA effects on root elongation exhibit a biphasic response modulated via crosstalk with auxin and ethylene pathways [22].

Transcriptomic analyses identified 2597 and 4412 genes consistently responsive to WD and WE, respectively, with 845 genes in common. The 845 shared genes likely represent a core ABA-dependent stress module of transcription factors, ROS scavengers, and signaling components that are activated whenever plants perceive severe water imbalance. In contrast, the WD-specific genes are enriched for functions that conserve water and protect photosynthesis (stomatal regulators, osmoprotectant biosynthesis, and secondary metabolism), whereas the WE-specific genes reflect hypoxia and remodeling programs (energy metabolism shift, cell-wall modification and hormone crosstalk, especially with ethylene). These patterns (core + stress-specific modules) have been observed in other soybean transcriptome comparisons and explain why ABA can trigger both shared defense mechanisms and divergent, context-dependent responses [13,23,24].

### 3.2. Discussion of Gene Regulation in Relation to ABA, WD, and WE

**Figure 8:**
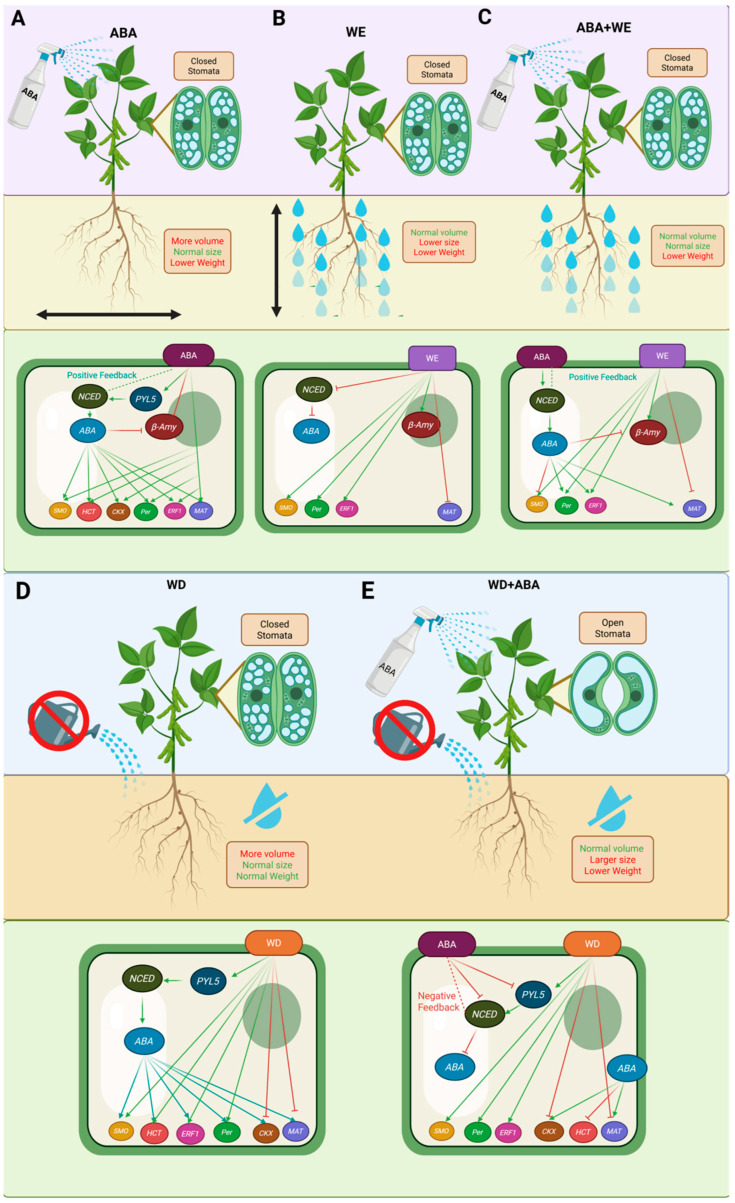
Representation of physiological, morphological, and molecular traits of Glycine max (Williams 82) under: control with ABA (**A**), isolated water excess (WE) (**B**), WE + ABA interaction (**C**), isolated water deficit (WD) (**D**), and WD + ABA interaction (**E**). Yellow dashed line = root size; gray roots = reduced dry mass; brown roots = normal dry mass. Green arrows = gene activation; red arrows = gene repression. Genes with dark green borders remain active; red borders remain inactive. Purple ABA = exogenous; navy blue ABA = endogenous.

In addition to physiological and morphological adjustments, our gene-level analysis provides a mechanistic view of how ABA modulates soybean responses to contrasting water stresses. As illustrated in Figure 8, ABA-responsive genes operate as a regulatory network that integrates hormonal crosstalk, redox balance, and metabolic adjustment, leading to opposite physiological outcomes in WD and WE.

*ERF1*. ERF family TFs have been repeatedly implicated in tolerance to abiotic stress. Constitutive overexpression of AtERF1 confers drought and salt tolerance with constitutively smaller stomatal apertures and reduced water loss [25,26]. In soybean, overexpression of *GmERF113* enhances drought tolerance and increases antioxidant enzyme activities, including Peroxydase [27]. Our finding that ERF1 is upregulated in WD and WE and induced by ABA suggests ERF1 as a nodal integrator of ethylene/ABA crosstalk and stomatal regulation in both stress contexts.

Peroxydase [25,26]. Multiple studies demonstrate that Peroxydases and APXs are key determinants of stress tolerance: APX knockouts (rice OsAPX2) reduce drought tolerance, while overexpression of Peroxydases improves drought resistance and lowers ROS accumulation [28,29,30]. The co-upregulation of Peroxydase with *ERF1* in our dataset is consistent with a mechanism whereby ABA/ERF signaling induces ROS-scavenging machinery to protect cellular components under both WD and WE.

*SMO* (spermine oxidase/sterol oxidase-like) showed divergent regulation, being induced in WD but suppressed in WE with ABA treatment. This pattern corresponds to documented functions of PAO-like enzymes in stress signaling. For instance, transgenic tobacco lines with altered PAO levels revealed that PAO-generated H_2_O_2_ acts as a second messenger in stress responses, although excessive production leads to cell death [31]. In rice, *OsPAO2* and *OsPAO6* were strongly induced by drought but repressed by ABA, aligning with our observation of ABA-mediated repression under WE [32]. A broader review of PAOs across species highlights their central role in ROS homeostasis and abiotic stress adaptation [33]. Furthermore, the dual capacity of PAO enzymes to generate H_2_O_2_ as both a signaling molecule and potential stressor has been well characterized [34]. Together, these results suggest that under WD, SMO-derived H_2_O_2_ could act as a beneficial acclimation signal, while its suppression under WE helps prevent excess ROS accumulation in hypoxic roots.

*NCED3*, encoding the key ABA biosynthesis enzyme, displayed context-dependent feedback regulation: it was repressed in WD + ABA, likely preventing excessive ABA accumulation when exogenous ABA was supplied, while it was upregulated in WE + ABA, reinforcing ABA synthesis under stress conditions. This dual regulation is supported by functional evidence: in soybean, transgenic lines overexpressing *AtNCED3* demonstrated elevated ABA levels, improved water-use efficiency, and higher yield under drought in greenhouse and field conditions [35]. Conversely, other studies suggest that *NCED* induction may be detrimental under flooding; for instance, NCED overexpression in soybean led to increased sensitivity to waterlogging and reduced yield due to oxidative stress [36]. Such divergent roles are consistent across species: Arabidopsis *NCED3* overexpressors show enhanced drought tolerance via ABA elevation [37], and rice *OsNCED3* overexpression similarly improves drought and salt tolerance [38]. Our data align with this model, positioning NCED3 as a molecular switch modulating ABA biosynthesis based on water stress context and explaining the opposing physiological outcomes observed in our experiments.

*MAT* (Methionine Adenosyltransferase), responsible for synthesizing SAM (the precursor of ethylene, polyamines, and methyl donors), was downregulated under both WD and WE, and further suppressed by ABA under WE. Proteomic analysis in soybean shows that SAM synthetase activity increases under drought but declines during flooding [39], supporting this stress-dependent regulation. This suggests that in WD + ABA, reduced SAM may limit ethylene synthesis, favoring stomatal reopening and higher gas exchange [40]. In contrast, under WE + ABA, stronger repression of *MAT* likely contributes to sustained ABA–ethylene dominance that suppresses photosynthesis. SAM also underpins multiple hormone and methylation pathways, positioning *MAT* as a critical metabolic pivot for balancing adaptive responses under contrasting water stress

*PYL5*, an ABA receptor, showed WD-specific regulation in our study. This observation is consistent with functional evidence from Arabidopsis and rice, where *PYL5* overexpression enhanced drought tolerance by promoting stomatal closure, reducing water loss, and improving root growth [41,42,43]. Mutants deficient in multiple *PYR/PYL* receptors display impaired ABA sensitivity and defective drought responses, reinforcing the central role of these receptors in ABA perception and signaling [44]. By contrast, the lack of *PYL5* induction under WE suggests that ABA perception is less dependent on *PYL5* during hypoxia responses, in line with the contrasting physiological roles of ABA under these conditions.

*HCT* (Hydroxycinnamoyl-CoA shikimate/quinate hydroxycinnamoyltransferase), a key enzyme in the phenylpropanoid pathway, displayed WD-specific regulation. Functional disruption of *HCT* in Arabidopsis severely reduces lignin and compromises vascular integrity and stress tolerance, underscoring *HCT’s* critical role in structural adaptation [45]. In contrast, the overexpression of *Camellia sinensis HCT* in Arabidopsis and tobacco conferred tolerance to abiotic and biotic stresses by increasing lignin and flavonoid precursors [46]. More recently, in soybean, alterations in proteins of the phenylpropanoid pathway, including those related to flavonoids and lignin biosynthesis, were identified as key determinants of drought tolerance through integrated proteomic and transcriptomic analyses in tolerant genotypes [47]. These findings support our observation of ABA-mediated induction of *HCT* during drought, suggesting enhanced lignification as a structural acclimation strategy, while its lack of induction under WE reflects distinct metabolic priorities when oxygen availability is limited.

*CKX* (cytokinin oxidase/dehydrogenase), an enzyme responsible for cytokinin degradation, showed WD-specific regulation in our study. This response is consistent with functional studies in Arabidopsis and rice, where *CKX* overexpression enhanced drought tolerance by reducing shoot growth and stimulating root proliferation [48,49]. Conversely, *ckx* mutants exhibit elevated cytokinin levels and increased stress sensitivity, confirming the protective role of *CKX* in abiotic stress adaptation [50,51]. In soybean, transcriptomic and genome-wide analyses also reported differential expression of *CKX* family members under drought, supporting its role in hormone balance during water deficit [52]. Together, these findings highlight *CKX* as a key component of the ABA–cytokinin crosstalk that modulates growth restraint and root system remodeling during drought acclimation.

β-amylase (*BAM*) is a key enzyme in starch degradation, but its role varies with stress type. In Arabidopsis, *BAM1* supports drought tolerance by fueling proline synthesis [53], and in sweet potato, *IbBAM1*.1 enhances drought and salt tolerance via ROS and osmotic balance [54]. Under flooding, transcriptome studies in legumes show BAM involvement in supplying sugars for anaerobic metabolism [55]. Consistent with this, we found that in soybean, BAM was not strongly induced by drought but was robustly regulated under water excess, suggesting a more prominent role in flooding tolerance than in drought.

*ERF1, Peroxydase*, and *SMO* emerged as the most consistent targets, integrating hormonal signaling with ROS management and showing strong potential for enhancing tolerance to both drought and flooding. Notably, while *SMO* was induced under both stresses, its repression in WE + ABA suggests a fine-tuned role in redox homeostasis that may depend on ABA–ethylene balance. Other genes, including *NCED3*, *PYL5*, *CKX*, *HCT*, *MAT*, and β-amylase, displayed more context-dependent regulation, indicating stress-specific or conditional contributions. Together, these results highlight *ERF1*, Peroxydase, and SMO as priority candidates for soybean improvement, while underscoring the need for deeper investigation into the context-dependent genes.

## 4. Materials and Methods

### 4.1. Greenhouse Experiment

A greenhouse experiment was conducted at Embrapa Soja (Londrina-PR) with soybean genotype Williams 82. Seeds were fungicide-treated, germinated and uniform seedlings were inoculated with *Bradyrhizobium japonicum* before transplanting into pots containing a sterilized soil–sand mixture. Plants were grown under controlled greenhouse conditions until the V3 stage [56], when three water regimes were applied water deficit (WD), water excess (WE), and control (C). Treatments were combined with the presence or absence of exogenous ABA in a 3 × 2 factorial design. ABA application was made of daily sprays for seven days [57]. Leaf samples were collected on the last day for RT-qPCR analysis.

### 4.2. Gas Exchange Analysis

Gas exchange measurements were performed using a portable photosynthesis system (LI-6400XT, LI-COR Biosciences, Lincoln, NE, USA). The flow rate inside the chamber was set to 400 μmol s^−1^, and the CO_2_ concentration in the reference chamber was controlled using the built-in CO_2_ injection system and maintained at 400 ppm, corresponding to ambient levels. Photosynthetically active radiation (PAR) was fixed at 1000 μmol photons m^−2^ s^−1^, a standard saturating light intensity for soybean and other C_3_ crops, ensuring that Net Photosynthesis were not limited by light availability but reflected the physiological status of the plants [58,59,60].

Net photosynthesis (A, μmol CO_2_ m^−2^ s^−1^), stomatal conductance (gs, mol H_2_O m^−2^ s^−1^), and transpiration rate (E, mmol H_2_O m^−2^ s^−1^) were recorded. Stomatal conductance readings were taken on the last day of treatment (7th day) between 13:00 and 14:00. Values above 0.2 mol H_2_O m^−2^ s^−1^ were considered typical for control plants, while values below this threshold indicated water deficit conditions [59,60].

### 4.3. Root Length and Dry Mass Measurements

Initially, the root length of soybean plants under control, WD, and WE conditions with and without exogenous ABA application was assessed using a graduated ruler. The dry mass of the roots was obtained by drying them in properly labelled paper bags in a 60 °C air-circulating oven for 2 days [61,62,63]. Subsequently, each sample was weighed using an analytical balance (Shimadzu AUY220 model).

### 4.4. Root Analysis by Image

Using the HP scanjet 6300c scanner and Safira software version 1.1 (http://www.cnpdia.embrapa.br/downloads/safira/ accessed on 1 October 2025), the volume (mm^3^) and surface area (mm^2^) of the soybean roots under control, WD, and WE conditions with and without exogenous ABA application were analyzed at the end of the experiment.

### 4.5. Identification of Genes Responsive to Water Deficit and Excess in rna-seq Libraries

To identify genes responsive to water deficit (WD) and water excess (WE), transcriptome data from BioProject PRJNA 324522 at NCBI were analyzed. Leaf samples from soybean cultivar Williams 82, subjected to control, WD, and WE treatments over seven days, were sequenced in biological triplicates using the Illumina Genome Analyzer, producing ~28 million single-end 100 bp reads, covering three times the genome size.

Raw reads were processed following best bioinformatics practices [64]. Cleaning was performed with Trimmomatic v0.36, retaining reads >40 bp and Phred ≥30 [65]. Quality was assessed before and after cleaning using FastQC v0.11.5 [66]. Cleaned reads were aligned to the *Glycine max* reference genome (Wm82.a2.v1) from Phytozome (https://phytozome.jgi.doe.gov/pz/portal.html#!info?alias=Org_Gmax accessed on 1 October 2025) using Hisat2 v2.1.0 [67]. Unique alignments were selected and PCR duplicates removed with Samtools rmdup v1.5 [68]. Transcript assembly used Stringtie v1.3.3 [69].

Differential expression was assessed using Gfold v1.1.4 for individual biological replicates [70] and EdgeR v3.11 considering biological variation [71], applying a false discovery rate (FDR) cutoff of 0.05. Only genes identified by both tools with |Log2FC| ≥ 1 were selected for further analysis.

### 4.6. Identification of Conserved Motifs in Promoter Regions Responsive to ABA and Abiotic Factors

For a more nuanced understanding of genes responses to abscisic acid (ABA) and various abiotic factors, an in-depth analysis of their promoter regions was performed. This process applied the RSATplant software tool [72] to isolate a 2000 base pair segment located upstream of each gene’s transcription start site (TSS).

This detailed study aimed to identify and enumerate ABA-responsive elements, or ABREs (ACGT sequence) [73], and elements responsive to flooding (represented by the sequences GCCCC, GGTTT, GCGCC, and AAACCA) [74]. These elements were sought within the genes’ promoter regions. To accomplish this, a custom script in Shell language was implemented, ensuring a comprehensive and efficient analysis.

### 4.7. System Biology

Gene annotation was performed using the W82.a2.v1 and W82.a4.v1 soybean genome annotations from Phytozome (https://phytozome.jgi.doe.gov/pz/portal.html, accessed on 25 September 2023), retaining only genes present in both versions to reduce annotation errors. Arabidopsis thaliana annotations were obtained from the Araport11 version in TAIR (https://www.arabidopsis.org/, accessed on 27 September 2023) when needed for functional inference. Gene copies, transcripts, and soybean orthologs were identified via the Persephone genome browser using Blastp (https://persephonesoft.com/, accessed on 27 September 2023). Metabolic pathways were assigned through the KEGG database (https://www.genome.jp/kegg/pathway.html, accessed on 27 September 2023), and differentially expressed genes were grouped using the Jvenn tool (http://jvenn.toulouse.inra.fr/app/example.html, accessed on 30 September 2023).

### 4.8. Gene Expression Analysis by RT-qPCR

Nine genes were selected from RNA-Seq data for RT-qPCR validation based on: (1) similar regulation profiles under WD and WE compared to the control, (2) functional annotation, and (3) the presence of at least two ABA-responsive motifs and one flooding-responsive motif in their promoters. Primers were designed using Primer3plus and evaluated with Multiple Primer Analyzer. Total RNA was extracted from bulked leaf samples with Trizol^®^ reagent, treated with DNase I, and tested for genomic DNA contamination using intronic β-actin primers. cDNA synthesis was performed with the SuperScript^®^ III First-Strand Synthesis System Kit, followed by verification of synthesis efficiency and absence of genomic DNA. RT-qPCR reactions were performed in triplicate with SYBR^®^ Green Master Mix on an Applied Biosystems 7900HT system. Relative gene expression was determined from three biological replicates using the 2^−∆∆Ct^ method, adjusted for primer efficiency [15], and normalized with β-actin and ELF1β as endogenous controls for WD and WE, respectively [75].

### 4.9. Statistical Analyses

The physiological and morphological data collected were subjected to analysis of variance (ANOVA). Mean comparisons were performed using Tukey’s test (*p* ≤ 0.05).

## 5. Conclusions

Our study identified a set of ABA-responsive genes that are differentially regulated under water deficit (WD) and water excess (WE), providing new insights into the molecular crosstalk between these contrasting stress conditions. Integration of transcriptomic analyses with RT-qPCR validation allowed the identification of robust and consistent genes. Among the nine genes selected from the RNA-seq library, seven genes showed the same regulation in RT-qPCR under water deficit (*PYL5*, *ERF*1, Peroxydase, *HCT*, *SMO*, *CKX*, and *MAT*), while five genes exhibited consistent regulation under water excess (β-amylase, *ERF1*, *Peroxydase*, *SMO*, and *MAT*). Considering both conditions simultaneously, only *ERF1* and Peroxydase were consistently upregulated in both techniques and under both stresses, highlighting them as promising molecular targets for developing soybean more resistant to both drought and flooding simultaneously.

Exogenous ABA exhibited context-dependent roles, acting as either a positive or negative regulator for different genes assessed in this study. However, for *ERF1* and Peroxydase, ABA consistently acted as a positive regulator, increasing their expression under both water deficit and excess, representing one of the key and most innovative findings of this article, and identifying reliable molecular targets for gene prospecting and soybean improvement. For the other candidate genes, a distinct ABA-mediated regulation pattern was observed, reflecting the complexity and specificity of ABA signaling under contrasting water stress conditions.

Therefore, this work provides a comprehensive molecular framework of ABA-mediated responses under WD and WE, highlighting consistent and reliable genes that can serve as molecular markers and potential targets for future functional studies and breeding programs, contributing to the development of soybean more resistant to both drought and flooding simultaneously.

## Figures and Tables

**Table 1:** Characterization of Selected Genes for RT-qPCR: Gene Copies, Transcripts, Orthologs in *Arabidopsis thaliana* and Enzymatic Codes.; Legend: Gene ID: Gene Identifier. SNC: Soybean gene copy number. %S: Similarity percentage. STN: Soybean transcription number. ATC: *Arabidopsis thaliana* gene copy number. ATN: *Arabidopsis thaliana* transcript number. na: Not applicable.

Gene ID	SNC	Copy Gene	%S	STN	Annotation	Enzyme Code	Orthologs	ATC	ATN	%S
Glyma.15G250100	na	na	93	1	*NCED3*	EC 1.13.11.51	na	na	na	na
Glyma.03G162700	na	na	na	1	*ERF1*	na	na	na	na	na
Glyma.02G254600	2	Glyma.14G061800	92	1/1	*HCT*	EC 2.3.1.133	AT2G39980	na	1	70
Glyma.02G261900	na	na	na	1	*PYL5*	Na	AT2G38310	na	1	69
Glyma.11G039400	2	Glyma.01G203400	94	1/1	*β-amylase*	EC 3.2.1.2	na	na	na	na
Glyma.18G045100	na	na	na	2	*SMO*	EC 1.5.3.17	AT1G65840	na	1	70
Glyma.19G223000	2	Glyma.03G226000	95	1/2	*MAN*	EC 3.2.1.78	na	na	na	na
Glyma.11G149100	na	na	na	1	*CKX*	EC 1.5.99.12	na	na	na	na
Glyma.18G258000	na	na	na	1	*MAT*	EC 2.3.1.115	na	na	na	na
Glyma.15G052600	na	na	na	1	*Peroxydase*	EC 1.11.1.7	na	na	na	na

## Data Availability

Data will be available upon request to the corresponding author.

## Supplementary Materials

The following supporting information can be downloaded at: https://www.mdpi.com/article/10.3390/plants14213245/s1. Supplementary File S1 (primer sequences) and Supplementary File S2 (Differentially expressed genes in response to water deficit and water excess).

## Abbreviations

The following abbreviations are used in this manuscript:

WD	Water Deficit
WE	Water Excess
ABA	abscisic acid
