# Supplementary material for: Molecular Mechanisms and Crosstalk Signaling in Soybean’s Response to Water Deficit and Excess: Implications for Stress Resilience and Productivity
Source: Plants (Basel). 2025 Oct 23;14(21):3245. doi: 10.3390/plants14213245 (PMC12608869; doi:10.3390/plants14213245)
Supplement: Supplementary file 1 [file plants-14-03245-s001.zip › Supplementary File 1 - Primer Sequences.pdf]

Supplementary File 1 - Primer Information and Sequences for RT-qPCR Validation. This table presents the primer identification and sequences for the selected genes used in the validation of RT-qPCR analysis, including both forward (F) and reverse (R) primers. The table includes the gene ID, primer efficiency, primer sequences (5'-3'), and the melting temperature (TM) of the primers. **Legend:** Gene ID - Gene identifier; TM - Melting temperature.

Gene ID	Annotation	Primer Efficiency	Primer Sequences (5´- 3)	TM
Glyma.15G250100	NCED3	86.0%	Primer F CCAGAAGCCTTACCTCAAAT<br>Primer R TCATGGTGGGTTCTTTCAAA	60<br>60
Glyma.03G162700	ERF1	94.6%	Primer F AAGTAAGCCATGGGAAGAAG3<br>Primer R GGTTGATCAGAGGGAACAAT	60<br>60
Glyma.02G254600	HCT	86.6%	Primer F CGATTCAGAAACTCAAAGCC3<br>Primer R TACTAAAGGAGAACAAGGCG3	60<br>60
Glyma.02G261900	PYL5	82.7%	Primer F TTTGTTTCGAATACGGGTTC3<br>Primer R ATCAAGGTCAGACGACAATG	60<br>60
Glyma.11G039400	*B*-Amylase	88.9%	Primer F CACTTCGCTCTTCAATTTCC<br>Primer R CCCATCTTTCCGTCTTAGAG	60<br>60
Glyma.18G045100	SMO	84.9%	Primer F TGAATTGGTGACTATCGTCC<br>Primer R TGTGGGTGTGTCCTACTTGT	60<br>60
Glyma.19G223000	MAN	90.2%	Primer F GTCGCCTACCTAGCTCATGT<br>Primer R TTCATAGGAAAATACCGCGT	60<br>60
Glyma.11G149100	CKX	95.5%	Primer F CTCAGTGTTGCAGCTAGAGG<br>Primer R TCAGGAGGGGAATTGTGGAT	60<br>60
Glyma.18G258000	MaT	90.2%	Primer F CCTACACTACCCTCCTCTCG<br>Primer R CATAGATTACTCAGTGCCCC3	60<br>60

Glyma.15G052600	Peroxidase 62	97.7%	Primer F AGAGCACTCTGCCCACAAA<br>Primer R TTGCTAATTTGAGGATTGGC	60<br>60
Glyma.15G050200	*B*-Actin	92.5%	Primer F TTCTGTCTTCTGCAAGTGGTG<br>Primer R GATCCCTCATCCATACATTTCAG	60<br>60
Glyma.02G276600	ELF1*B*	94.5%	Primer F GTTGAAAAGCCAGGGGACA<br>Primer R TCTTACCCCTTGAGCGTGG	60<br>60
